# Internal Consistency of Sway Measures via Embedded Head-Mounted Accelerometers: Implications for Neuromotor Investigations

**DOI:** 10.3390/s21134492

**Published:** 2021-06-30

**Authors:** Andrew P. Lapointe, Jessica N. Ritchie, Rachel V. Vitali, Joel S. Burma, Ateyeh Soroush, Ibukunoluwa Oni, Jeff F. Dunn

**Affiliations:** 1 Hotchkiss Brain Institute, University of Calgary, Calgary, AB T2N 4N1, Canada; joel.burma@ucalgary.ca (J.S.B.); ateyeh.soroush@ucalgary.ca (A.S.); ibukunoluwa.oni1@ucalgary.ca (I.O.); 2 Integrated Concussion Research Program, University of Calgary, Calgary, AB T2N 4N1, Canada; 3 Department of Radiology, Cumming School of Medicine, University of Calgary, Calgary, AB T2N 4N1, Canada; 4 Department of Clinical Neurosciences, Cumming School of Medicine, University of Calgary, Calgary, AB T2N 4N1, Canada; 5 Schulich School of Engineering, University of Calgary, Calgary, AB T2N 4N1, Canada; jessica.ritchie@ucalgary.ca; 6 Department of Mechanical Engineering, University of Iowa, Iowa City, IA 52242, USA; rachel-vitali@uiowa.edu; 7 Sport Injury Prevention Research Centre, Faculty of Kinesiology, University of Calgary, Calgary, AB T2N 4N1, Canada

**Keywords:** accelerometer, sway, reliability, balance, multimodal, inertial measurement unit (IMU)

## Abstract

Accelerometers are being increasingly incorporated into neuroimaging devices to enable real-time filtering of movement artifacts. In this study, we evaluate the reliability of sway metrics derived from these accelerometers in a standard eyes-open balance assessment to determine their utility in multimodal study designs. Ten participants equipped with a head-mounted accelerometer performed an eyes-open standing condition on 7 consecutive days. Sway performance was quantified with 4 standard metrics: root-mean-square (RMS) acceleration, peak-to-peak (P2P) acceleration, jerk, and ellipse area. Intraclass correlation coefficients (ICC) quantified reliability. P2P in both the mediolateral (ICC = 0.65) and anteroposterior (ICC = 0.67) planes yielded the poorest reliability. Both ellipse area and RMS exhibited good reliability, ranging from 0.76 to 0.84 depending on the plane. Finally, jerk displayed the highest reliability with an ICC value of 0.95. Moderate to excellent reliability was observed in all sway metrics. These findings demonstrate that head-mounted accelerometers, commonly found in neuroimaging devices, can be used to reliably assess sway. These data validate the use of head-mounted accelerometers in the assessment of motor control alongside other measures of brain activity such as electroencephalography (EEG) and functional near-infrared spectroscopy (fNIRS).

## 1. Introduction

Imbalance is associated with aging [1,2], Parkinson’s disease [3], multiple sclerosis [4,5], Alzheimer’s disease, concussion, and many other clinical conditions [6]. Therefore, evaluations of postural stability such as the Berg Balance Scale (BBS), Balance Error Scoring System (BESS), and Timed-Up-and-Go (TUG) test have become an integral part of the clinical screening process. Increasingly, inertial measurement units (IMUs) are also being utilized to quantify sway during these balance assessments in an effort to reduce subjectivity [5,7,8]. In its simplest configuration, an IMU is a micro-electromechanical system (MEMS) containing a triaxial accelerometer and angular rate gyroscope that measure linear acceleration and angular velocity, respectively. Pertinent to this work, an abundance of literature has demonstrated the validity of the measurements provided by IMUs including the accelerometry data [4,9,10,11,12]. It is proposed that IMUs can obtain accelerometry data, measuring both linear acceleration and angular velocity, respectively.

Historically, vestibular impairment in static balance is assessed with force plates, which record the center of pressure (COP) displacements from equilibrium. The data provided by this technology are used to develop stabilograms, which are commonly analyzed in posturography [2]. Further, force plates are considered the gold standard assessment technology within balance research [13]; however, the cost and size of force plates inhibit their adoption for clinical use. Consequently, IMUs represent an inexpensive and portable alternative for providing sway metrics associated with balance [14].

In the literature, these devices are predominately attached to the waist/lower back, which serves as a proxy for the body’s center of mass (COM) [15]. In a systematic review of 47 articles utilizing IMUs to assess standing balance, Ghislieri et al. [16] found 80.9% of studies positioned the device at the COM. The metrics extracted from the accelerometry data provided by the waist-mounted IMUs display similar reliability to and good correlation with the metrics provided by force plates during static balance tasks [4,9,10,11,12]. Similarly, several studies further demonstrated the potential of accelerometry data provided by IMUs attached to the upper thorax (i.e., the trunk) as well [17,18].

Though considerably less common, several studies also investigated the ability of head-mounted IMUs to quantify sway. Grafton et al. [6] and Salisbury et al. [19] both evaluated the reliability of accelerometry-derived sway measures for a static standing balance. Specifically, Grafton et al. [6] demonstrated low session-to-session variability in sway power derived from accelerometry data from head-mounted IMUs, whereas Salisbury et al. [19] found consistent test–retest reliability in accelerometry-derived normalized path length between head- and waist-mounted IMUs. While their results support the utility of head-mounted IMUs, sway power and normalized path length are relatively uncommon sway metrics in the literature utilizing waist-mounted devices. By contrast, Lubetzky et al. [20] examined the root-mean-square (RMS) of accelerometry data collected by a virtual reality headset. While RMS is the one of the most common sway metrics derived in studies utilizing waist-mounted IMUs [7,11,12,16,17,21,22,23,24,25,26], participants were subject to a visual weighting paradigm. The vision condition was used to assess head kinematics in relation to moving images [20]. Together, these studies do not reflect typical balance assessments performed with waist-mounted IMUs.

With the increase in integrated accelerometers in neuroimaging systems, head-mounted IMUs are an attractive option for the collection and analysis of sway data with brain functionality. However, the potential of these embedded accelerometers has been limited to the detection of motion artifacts in neuroimaging data. These accelerometers are ideally situated for the development of a multimodal (i.e., the simultaneous use of both neuroimaging and accelerometry) study protocol, which would enable investigations into the relationship between modalities. While functional near-infrared spectroscopy (fNIRS) and electroencephalography (EEG) have been combined to evaluate gait and standing balance (for a review, see [27,28,29]), the relationship between brain functionality and balance has not been examined using multiple neuroimaging modalities. This new multimodal experimental design could have huge implications for clinical populations suffering neurological conditions affecting balance. However, there is a paucity of studies comparing the reliability of commonly used sway measures derived from accelerometry data provided by head-mounted IMUs. Moreover, an in-depth investigation into which accelerometry-derived metrics, if any, are best suited for this application has yet to be demonstrated. Thus, the aim of this study was to determine the reliability over one week of sway metrics derived from a head-mounted accelerometer embedded in an integrated neuroimaging system.

## 2. Materials and Methods

### 2.1. Participants

A convenience sample of 10 healthy controls (6 females, 4 males) aged 24.1 ± 3.1 years (range: 20–30 years) were recruited to participate in this study (height: 1.70 ± 0.12 m, weight: 65.2 ± 16.9 kg). Individuals who self-reported a concussion history were included in the study if they were fully recovered and more than six months had passed since their most recent concussion at the time of testing. Daily questionnaires were used to gauge participants’ mental and physical health. Additionally, consumption of food, caffeine, alcohol, prescription or recreational drug use, and recent physical activity were self-reported. Participants were outfitted with appropriately sized integrated fNIRS and EEG embedded head caps based upon (1) nasion to inion distance (37.55 ± 2.11 cm), (2) distance between the right to left preauricular points (34.60 ± 2.07 cm), and (3) head circumference (56.0 ± 2.4 cm). All subjects provided written informed consent. Demographics are presented in Table 1.

### 2.2. Testing Protocol

**Figure 1:**
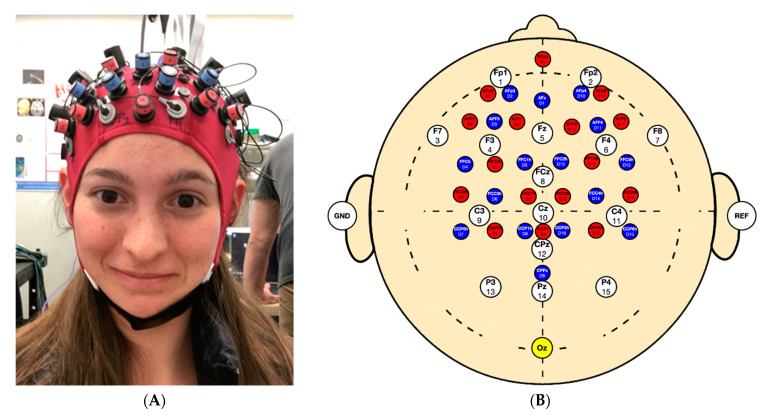
(**A**) Cap setup showing experiment setup and (**B**) montage demonstrating the position of the accelerometer in yellow at Oz.

Each of the 10 subjects were tested daily over 7 consecutive days for a total of 70 measures. Data were collected during a multimodal study integrating fNIRS, EEG, and electrocardiography (ECG). Participants performed 4 tasks in serial order: an 8 min seated rest, 11.5 min 2-back, 4 min of finger tapping, and 8 min of standing. The standing task, the focus of this investigation, was performed on a firm surface with shoes on and eyes open staring straight ahead at a wall. Participants stood with their feet in a comfortable, self-selected position with arms at their sides. Testing was conducted in a quiet, dimly lit room. Head motion data were acquired using a NIRSport2 IMU (NIRx Medical Technologies, CA, USA), which provided 3D measurements of linear acceleration, angular velocity, and magnetic field strength. The IMU was centered over “Oz” in accordance with the 10–20 system (i.e., over the participants occipital lobe) via the embedded fNIRS EEG system (Figure 1A). In Figure 1B, we demonstrate the 10–20 montage which shows EEG electrode position (in white), fNIRS sources (red) detectors (blue), and accelerometer (yellow).

### 2.3. Data Processing

Data were sampled at 100 Hz for the entire testing protocol, where only data from the standing task were analyzed. Three consecutive minutes of stable linear acceleration were extracted during the standing task. Data were extracted between minutes 3 to 6 for most recordings; however, for 6 recordings, times were manually chosen as they showed artifacts during that time frame. One recording was rejected from the final dataset due to an equipment error leaving n = 69 measures, each containing 18,000 data points. Due to this missing data point, data from the 7th measure for all participants were dropped in order to properly calculate ICC values resulting in a final sample of n = 60 measures.

To account for tilt caused by variation in head shape, data were projected onto the subject’s anatomical frame. A full description of the tilt-correction method is described in Appendix A. Finally, the data were low-pass filtered with a 7th order infinite impulse response (IIR) elliptical filter using a cutoff frequency of 10 Hz.

Four types of sway metrics were calculated from the 3 min of cleaned and trimmed standing data. Root-mean-square (RMS) acceleration is frequently used in waist- and trunk-mounted IMU balance assessments [5,7,9,11,12,17,21,30]. It was calculated in the mediolateral (ML) and anteroposterior (AP) directions, and as a total in the transverse plane. Peak-to-peak (P2P) acceleration is the range of acceleration values [2,9,24,31,32] and was calculated for the ML and AP data only. Next, RMS jerk was derived from the linear acceleration in all three axes as described by Johnston et al. [33]. The numerical derivatives for each direction were computed according to Diop et al. [34]. Jerk is the time rate of change of acceleration, which describes the smoothness of sway. Low jerk indicates coordinated motion while greater jerk describes more disjointed movements. Jerk was chosen because it is commonly implemented in waist-mounted IMU studies, though it is frequently reported incorrectly with units of m^2^/s^5^ [5,10,16,35]. While the definition for calculating jerk reported by Johnson et al. [33] is accurate, we were unable to find other studies that utilized it for assessing a static balance task. Lastly, ellipse area was calculated in the transverse plane with 95% confidence [2,4,21,32]. Ellipse area quantifies the magnitude of the subject’s change in direction while balancing. A greater ellipse area suggests more severe changes in direction to correct for deviations from equilibrium. All measures used in our analysis are summarized in Table 2. All data processing was done with custom scripts in MATLAB (MATLAB 2019b, MathWorks, Natick, MA USA).

### 2.4. Statistical Analysis

Measures of reliability were calculated through intraclass correlation coefficients and interpreted using published guidelines [36]. Scores below 0.5 were characterized as poor, between 0.5 and 0.75 as moderate, between 0.75 and 0.9 as good, and above 0.9 as excellent.

ICC calculations and their 95% confidence intervals were computed, using custom scripts in R [37] (v4.0.3) with the psych package, based on a mean-rating (*k* = 3), absolute-agreement, 2-way mixed effects model (i.e., ICC (3, k)). Figures were created using ggplot2 [38].

## 3. Results

Jerk demonstrated the greatest reliability (ICC = 0.95), while AP-P2P demonstrated the poorest reliability (ICC = 0.67). ICCs for each sway metric, and confidence intervals, are reported in Table 3 with an accompanying illustration in Appendix B. Peak-to-peak metrics in both the ML and AP planes demonstrated moderate reliability with ICC values of 0.65 and 0.67, respectively. The remaining metrics all demonstrated ICC values above 0.75 and were classified as either “Good” or “Excellent” [36].

## 4. Discussion

Accelerometers are increasingly being incorporated into neuroimaging equipment to filter movement artifacts known to contaminate imaging data. These embedded accelerometers represent an opportunity to simultaneously evaluate sway in conjunction with brain activity. It is important to note that sway metrics derived from head-mounted accelerometers are highly correlated with sway metrics derived from waist-mounted accelerometers [19]. Thus, the purpose of this study was two-fold. First, we sought to establish the reliability of head-mounted accelerometers. Second, we sought to determine how sway metrics could be included into a multimodal study with brain activity. It should also be noted that a limitation of this study, and of accelerometers as tools to assess sway, is that mean velocity was not pursued as a sway metric. Despite its clinical significance, extracting accurate and precise estimates of velocity from multiple minutes of acceleration data is not possible with our current experimental design.

The reliability of AP-RMS acceleration (ICC = 0.76) was consistent with previous studies using waist-mounted accelerometers like Reynard et al. [17] and Kosse et al. [25], who reported ICC values of 0.83 and 0.70 to 0.95, respectively. Jerk demonstrated the highest reliability among our sway metrics (Table 3). This finding is in contrast to observations made by Williams et al. [26], who found jerk and RMS acceleration metrics to be more variable across their study. This contradiction is possibly due to the multiple conditions included in their study as opposed to the single standing task analyzed in this study.

Low ML reliability could be attributed to inconsistent test conditions and fatigue from standing. Upcoming studies will control for factors that could have negatively impacted reliability measures (e.g., by removal of participant’s shoes). Although outside the scope of this investigation, future work should examine potential confounds (e.g., sex, age, concussion history), all of which could impact the reliability of sway metrics.

In general, there is some difficulty in comparing the metrics derived in this study to those presented in prior works for several reasons. First, the lack of uniformity in task selection is a significant hurdle to new research in the field. To the best of our knowledge, reliability data for the remaining metrics listed in Appendix B have yet to be reported during an eyes-open standing task. Next, some studies employ metrics provided by proprietary software (i.e., a black box) such as APDM’s Mobility Lab [7] or SWAY Balance [18]. Additionally, the definition of jerk used in many studies (e.g., [3,4,5,10]) is actually a cost function proposed by Flash and Hogan [39] to minimize jerk when optimizing an unconstrained arm trajectory. However, it is likely that the result provided by the cost function is moderately to highly correlated with average jerk.

**Figure 2:**
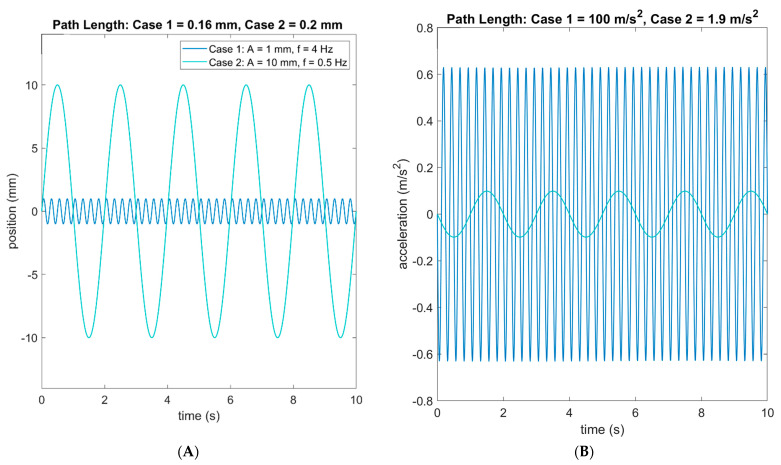
Hypothetical position (**A**) and acceleration (**B**) profiles.

Furthermore, metrics calculated with position data do not necessarily translate to those calculated with acceleration data. For example, many studies derive a path length using COP position data from force plates, which represent the total distance traveled by the center of gravity over the course of a trial. Some studies then normalize by trial time, essentially transforming this into an average speed for the center of gravity (e.g., Salisbury [19]). As aforementioned, this position-based metric does not necessarily translate to acceleration data, which is why it was not calculated for this study. To illustrate this point, consider the following limiting case. Figure 2A shows two hypothetical sinusoidal profiles of a subject’s COP in one direction, and Figure 2B shows the corresponding acceleration profiles. Oscillations are shown for two cases, the first with an amplitude of 1 mm at a frequency (f) of 4 Hz and the second with an amplitude of 10 mm at a 0.5 Hz frequency. Using the position data, the path length for Case 2 is larger than that for Case 1, which is consistent with the larger amplitude oscillations. However, using the acceleration data, the path length for Case 1 is significantly larger than that for Case 2, thereby contradicting the result derived using the position data. This type of discrepancy likely contributes to why the greatest correlation coefficient for the eyes-open condition between these two path lengths reported by Whitney et al. [9] was 0.57. Without considering the frequency content of the data, the acceleration path length is potentially misleading.

In the same vein, it is also likely that the power spectrum for the frequency content in the position data will be different from the power spectrum for the frequency content in the acceleration data. It is the nature of differentiation that higher frequencies are emphasized, which suggests the power spectrum for the acceleration data will be concentrated at higher frequencies as compared to the power spectrum for the position data. In fact, the acceleration frequency content was consistently higher than the position frequency content reported by Mancini et al. [3], and this difference was statistically significant for the untreated Parkinson’s group. This is particularly relevant to this study given that the frequency content at a person’s center of mass is shifted to higher frequencies than their center of gravity, which suggests the frequency content at the head could be shifted even higher [15]. This shift in frequency content also becomes important given that most studies low-pass filter both position and acceleration data prior to calculating metrics, some of which will be more sensitive to this filtering than others. Ideally, the frequency cutoff should be sufficiently high to avoid discarding valuable acceleration data, though an appropriate frequency cutoff is somewhat disputed. Reynard et al. [17] determined a cutoff of 30 Hz was sufficient, whereas Martinez-Mendez et al. [32] suggested a much lower cutoff of 5 Hz was sufficient, though some studies use a cutoff as low as 1.25 Hz [19].

Due to our inclusion of other modalities in our study protocol (i.e., fNIRS, EEG, and ECG), a minimum task length of 8 min was required. Our task length differed substantially from previous reports. In their detailed review, Ghislieri et al. [16] highlighted the average test duration was rarely over 30 s. In addition to the length of our standing task, brain imaging studies often require multiple measurements spanning several days. To our knowledge, reliability with more than two measures has yet to be reported. Despite variations in previous study protocols, we observed reliability indices in all sway metrics over our 6 days of testing which were comparable to prior reports with waist-mounted [17,25] accelerometers.

## 5. Conclusions

Our study has shown that data from a head-mounted accelerometer can provide reliable metrics of sway. This validates the use of head-mounted accelerometers in future multimodal neuroimaging studies. The combination of neuroimaging modalities with sway metrics will help our understanding in clinical conditions known to include neuromotor deficits.

## Figures and Tables

**Table 1:** Demographics.

	Sex	Age	Weight (kg)	Height (m)	BMI (kg/cm^2^)	Nasion to Inion (cm)	Preauricular Point to Preauricular Point (cm)	Head Circumference (cm)
1	F	23	48	1.60	18.75	39.0	37	56.0
2	F	30	61	1.55	25.39	35.0	34	55.0
3	F	24	50	1.58	20.03	39.0	36	57.0
4	M	27	96	1.88	27.16	42.0	36	60.0
5	F	20	50	1.64	18.59	37.0	34	52.5
6	M	21	64	1.63	24.09	37.0	33	55.0
7	M	24	77	1.83	22.99	38.0	38	58.5
8	F	21	58	1.78	18.31	37.5	32	56.0
9	F	26	58	1.63	21.83	35.0	34	52.5
10	M	25	90	1.88	25.46	36.0	32	57.0

**Table 2:** Sway metrics.

Metric	Description	Directions	Units
RMS	Sway magnitude	ML, AP, Transverse Plane	$\frac{m}{s^{2}}$
P2P	Range	ML, AP	$\frac{m}{s^{2}}$
Ellipse Area	Direction change	Transverse Plane	$\frac{m^{2}}{s^{4}}$
Jerk	Smoothness of motion	Resultant Jerk from ML, AP and V data	$\frac{m}{s^{3}}$

**Table 3:** Reliability table.

Metric	ICC	Lower Bound	Upper Bound	F	df1	df2	*p*	Classification
Ellipse Area	0.78	0.52	0.92	4.44	8	48	>0.001	Good
Anteroposterior Root Mean Square Acceleration	0.76	0.48	0.92	4.10	8	48	0.001	Good
Total Root Mean Square Acceleration	0.84	0.66	0.95	6.28	8	48	>0.001	Good
Mediolateral Root Mean Square Acceleration	0.79	0.55	0.93	4.71	8	48	>0.001	Good
Anteroposterior Peak-to-Peak	0.67	0.30	0.89	3.05	8	48	0.007	Moderate
Mediolateral Peak-to-Peak	0.65	0.24	0.88	2.83	8	48	0.012	Moderate
Jerk	0.95	0.90	0.98	21.19	8	48	>0.001	Excellent

**Figure A1:**
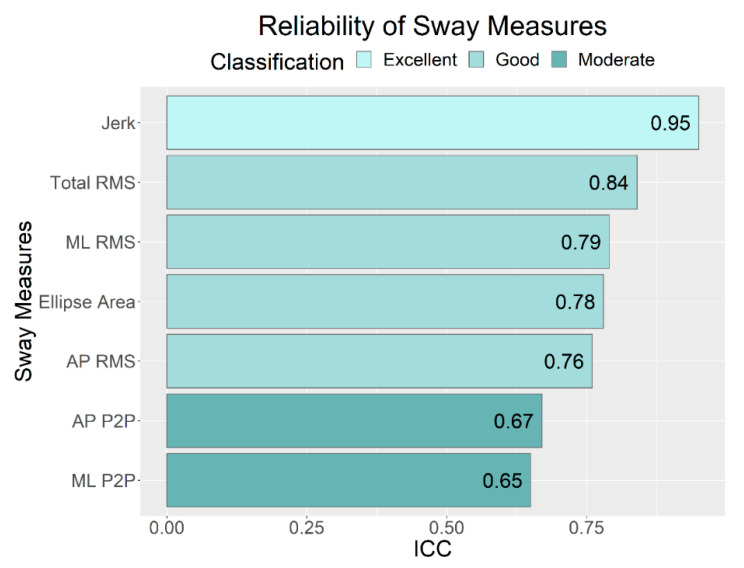
Visual representation of the reliability for each sway metric.

## Data Availability

Data can be made available on request to the correspondent author’s email with appropriate justification.

## Appendix A. Tilt-Correction Method for Linear Acceleration of a Single Recording

A unit vector, $\hat{v},$ describing the vertical direction of the IMU, was derived from the average linear acceleration, ${\bar{a}}_{measured}$, over the entire standing task.

$$\hat{v}=\left[\begin{array}{ccc} \overset{-}{ML}, & \overset{-}{AP}, & \overset{-}{V} \end{array}\right]$$

$$\hat{v}=\frac{{\bar{a}}_{measured}}{\left|{\bar{a}}_{measured}\right|}$$

The angle between the accelerometer’s vertical axis and the subject’s anatomical vertical axis was found using the dot product equation where

$$z=\left[\begin{array}{c} 0,0,1 \end{array}\right]$$

A unit vector $\hat{k}$ describing the axis about which to rotate the data was derived from the cross product of the accelerometer’s vertical axis and the subject’s anatomical vertical axis.

$$k=z\times \hat{v}$$

$$\hat{k}=\frac{k}{\left|k\right|}$$

This was used to define the skew-symmetric cross-product matrix K.

$$K=\left[\begin{array}{ccc} 0 & -\hat{k}\left(3\right) & \hat{k}\left(2\right)\\ \hat{k}\left(3\right) & 0 & -\hat{k}\left(1\right)\\ -\hat{k}\left(2\right) & -\hat{k}\left(1\right) & 0 \end{array}\right]$$

The direction cosine matrix R was found using Rodrigues’ rotation formula,

$$R=I+\left(\begin{array}{c} \sin\theta \end{array}\right)K+\left(\begin{array}{c} 1-\cos\theta \end{array}\right)K^{2}$$

where I is a 3 × 3 identity matrix. Finally, the accelerometer data were transformed by multiplying the transpose cosine matrix with our acceleration vector.

$$a_{i,new}=R^{T}a_{i,measured}$$
